# Eating disorder symptoms and weight pressure in female rowers: associations between self-concept, psychological well-being and body composition

**DOI:** 10.1186/s40337-024-01033-9

**Published:** 2024-06-14

**Authors:** Beñat Larrinaga, Erika Borrajo, Iker Muñoz-Perez, Itziar Urquijo, Ana Garcia-Rodríguez, Ane Arbillaga-Etxarri

**Affiliations:** 1 Deusto Healh-PASS, Physical Activity and Sport Sciences Department, Faculty of Education and Sport, University of Deusto, Bilbao, Spain; 2 Deusto Sport and Society, Physical Activity and Sport Sciences Department, Faculty of Education and Sport, University of Deusto, Bilbao, Spain; 3 Deusto Physical TherapIker, Physical Therapy Department, Faculty of Health Sciences, University of Deusto, Donostia-San Sebastián, Spain

**Keywords:** Weight pressures in sport, Female, Rowing eating disorder symptoms, Prevalence, Psychological well-being, Self-concept, Body composition

## Abstract

**Background:**

Female rowers may be at risk of eating disorders and high weight pressure.

**Aim:**

The purpose of the study was to investigate the prevalence of disordered eating symptoms and weight-related pressure and the associations with self-concept, psychological well-being, socio-demographic data, experience, performance level and body composition in female fixed-bench rowers.

**Methods:**

Female rowers (n = 208; age ranged mean ± SD 23.6 ± 6.5 years) completed the SCOFF scale, Weight-Pressures in Sport-Females (WPS-F), Physical Self-Concept Questionnaire and the Ryff scales of psychological well-being and provided information on their experience and level of competition. In a subgroup of 115 athletes, body composition was assessed using bioimpedance.

**Results:**

It was found that 42.3% of the athletes scored ≥ 2 on SCOFF and mean ± SD value of WPS-F score was 3.65 ± 0.82. Stepwise regression analysis revealed that self-concept of strength and pressure from teammates and the uniform were associated with higher ED symptoms, whereas better psychological well-being in terms of autonomy, self-concept of attractiveness, and age were protective factors for ED symptoms. BMI, athletes’ physical condition, strength, and experience were associated with more weight-related pressure and better self-concept of attractiveness and physical well-being of autonomy were significantly associated with less pressure. In body composition analysis, higher extra cellular water, self-acceptance, and physical condition were associated with more weight-related pressure in female rowers, being attractiveness and the environmental mastery protective elements.

**Conclusions:**

The prevalence of ED symptomatology and weight-related pressure are high in female fixed bench rowing. The psychological factors of well-being and self-concept, team environment, body image concerns and body composition analysis should be considered to promote healthy eating behaviours in female rowers.

**Supplementary Information:**

The online version contains supplementary material available at 10.1186/s40337-024-01033-9.

## Abstract (plain language summary)

Athletes may be at increased risk of developing disordered eating behaviours that, if persistent, may lead to a diagnosis of an eating disorder. This can affect their health in the short and long term. The risk factors are many, and perceived weight-related pressures and psychological attitudes towards body image, appearance and concerns are less well understood. Female rowers are at high risk for eating disorder symptoms, and athletes' self-concept of strength and pressure from teammates and the uniform were associated as negative factors for eating disorder symptoms. On the other hand, better psychological well-being in terms of autonomy, self-concept of attractiveness and age were protective factors for eating disorder symptoms. Weight pressure was associated with higher body mass index, water retention, physical condition, strength and experience, whereas better self-concept of attractiveness and physical well-being were significantly associated with less pressure. Weight pressure and psychological attitudes towards body image have the role of protective/facilitating factors against the development of eating disorders. They should be taken into account when designing prevention and intervention programmes for athletes, coaches, parents and technical staff.

**Supplementary Information:**

The online version contains supplementary material available at 10.1186/s40337-024-01033-9.

## Introduction

Eating pathology spans from disordered eating (DE) psychopathology or behaviour to a clinical diagnosis of an eating disorder (ED). DE is a serious mental-health condition in which the physical and psychological demands of the high levels of competition from each sport disciplines may increase athletes’ desire to achieve a specific and ideal body shape, composition, aesthetic, or weight. Thus, athletes can show an increased risk of developing physical overtraining, high pressure related to weight loss, and aggressive practices or dysfunctional eating behaviours patterns (such as intentional fasting, dieting, water loading and salt manipulation, vomiting, and use of laxatives and/or diet pills [1]. On the other hand, ED are persistent clinical alterations diagnosed according to Diagnostic and Statistical Manual of Mental Disorders (DSM-5) criteria which can be led by a continuum DE behaviour and nowadays there has been growing interest in their impact on sports performance [2].

Despite there is a large literature, the prevalence and the risk factors of ED psychopathology differs widely between studies depending on the sport, age, athlete type, or level of competition [3]. Studies also vary in their measurement, being unclear whether the psychological incidence is higher or lower than in non-athletes, and whether certain groups of athletes or type of sport are susceptible to or protected from ED [4].

Indeed, ED in athletes can affect their health and performance, as well as their future life outside of sport. In terms of performance, and physiological consequences, poor nutrition can lead to impaired metabolism, reduced bone density, amenorrhoea, osteoporosis and a weakened immune response, thereby increasing the risk of injury and illness [5]. These adverse health effects can affect athletic performance in a number of ways, including reduced recovery leading to premature decline in physical performance and impairment of optimal muscle mass and function [5]. Psychologically, in the short term, the stress and anxiety associated with DE can impair concentration, reaction time and decision-making, further hindering athletic success [5]. In the long term, the impact on mental wellbeing extends beyond the sporting career. Anxiety, depression and an unhealthy relationship with food can continue to affect personal and professional life [5].

The main factors associated with DE and ED are biological, psychological, sociocultural, and gender-based [6, 7]. Although ED in sport occurs in both genders [8], multiple studies indicate that women have a higher ED psychopathology and higher prevalence of EDs [9, 10] and a recent systematic review concluded that age did not moderate DE in women athletes [4]. In the general female population, it is known that satisfaction with body image is protective for DE due to the positive association with body functionality appreciation, self-esteem, self-compassion, and psychological well-being [11–13]. At the same time, negative physical self-concept may be attributed, especially in women, to the excessive expectations of conforming to societal norms [14] and achieving sporting excellence [15]. In female athletes, the dissatisfaction with body image is one of the strongest predictors of DE [16]. However, psychological attitudes towards body image, appearance and concerns have been less studied [12] in athletes participating in weight-sensitive sports, so the evidence is contradicting and more research is needed [12, 17, 18].

In general, athletes are more susceptible to weight pressure to lose or maintain a particular body composition, shape or weight in order to achieve optimal performance for the sport-specific characteristics or social ideals of appearance [12, 19]. The pressures to body image or composition to achieve optimal performance may be due to internal factors such as an athlete’s self-concept, psychological well-being and personality traits, or external factors such as elements of the sport environment (coaches, sport system or teammates) or social pressure, fans, media [20]. In particular, the pressure from a coach has been identified as one of the main risk factors for body image concerns and DE in athletes [21, 22] and the teammates also may be an important source of pressure that negatively influences athletes’ eating attitudes [23]. In turn, psychological well-being, body image self-concept and type of sport (aesthetic and lean) are related to body image concerns and dissatisfaction [4]. Petrie and Greenleaf’s model posits that sociocultural and sport specific pressures lead to ED symptoms through the internalisation of stereotypical body ideals, body dissatisfaction and unhealthy eating behaviours [24]. Therefore, assessing these factors can be a valuable tool in the early detection of individuals who may be at risk of developing eating and weight-related problems [25], particularly amongst women in weight-sensitive sports.

Among aerobic sports, the Sundgot-Borgen’s [26] classification includes rowing as a weight-dependent sport. Some categories require weight restrictions and, as an aesthetic or endurance sport, athletes seek to achieve and maintain a specific body weight and aerobic power to enhance sports performance or meet aesthetic ideals or weight limits [27–30]. In Olympic rowing modality, the motivation to reduce or maintain a low weight is based on the competitive advantage gained when an athlete is assigned to a lower weight category [31]. As a result, athletes often compete in a weight class lower than their natural body weight [32, 33]. In the fixed seat modality, there is no weight-class restriction but there are several weight-dependent characteristics. The crew is made up of 13 rowers and a skipper, and they compete in a 200-kg boat on the open sea and in the bays and rivers of the Cantabrian Sea. The aim of this sport is to shape the athlete’s body composition to be predominantly low in body fat, high in muscle mass and as light as possible [34] as a fundamental role in performance [35, 36]. The power-to-weight ratio is a predictor of performance because it directly affects the hydrodynamics of navigation [27] along with aerobic capacity, VO2 max and maximal strength [37, 38]. In fact, reducing the weight of the boat and distributing the crew appropriately according to the boat model and the conditions of the course reduce the boat’s water resistance, resulting in greater speed and performance [35]. This can increase weight-related pressure in athletes collectively and consequently, increase the risk of pathogenic weight control behaviours and the susceptibility to clinical ED [2]. Some studies have addressed the likelihood of DEs in female rowers [39–41] and in lightweight class an increased risk of athletes using extreme weight-loss tactics has been reported, as well as a higher prevalence of ED and short and long-term physical and psychosocial negative impact [6, 42–44]. However, there is currently limited research on this topic [43] and to the best of our knowledge, this has never been studied in fixed-bench rowing [45].

Therefore, the primary aim of this study is to show the prevalence of ED symptomatology and the weight-related pressure in female fixed-bench rowers. The secondary aim is to describe the correlates of the risk of ED symptomatology and the weight-related pressure regarding to self-concept, psychological well-being, socio-demographic data, experience, performance level and body composition. It was hypothesized that: (a) A high prevalence of ED symptomatology and weight-related pressure would be observed in female fixed-bench rowers; (b) ED symptomatology would be associated with socio-demographic data, weight-related pressure, self-concept, psychological well-being, body composition; and (c) Weight-related pressure would be associated with socio-demographic data, self-concept, psychological well-being, and body composition.

## Methods

### Design and study population

Female fixed-bench rowers from all clubs (23 in total) in collaboration with the main rowing sporting entities and institutions (federations, clubs, and the athletes) were recruited to this cross-sectional study. Athletes participated on the two competitive levels allocated on the Cantabrian Sea (north coast of Spain; The Basque Country, Cantabria and Galicia). The measurements were taken during the month of June 2021, before the start of the official season. Inactive rowers, underage rowers and other coastal regions of the Iberian Peninsula were excluded.

A convenience sample of 208 athletes was included, representing 45% of the total number of athletes practising this sport in an official and federated way. The age ranged 23.6 ± 6.5 years and 22% and 78% of them participated in the first and second competition level, respectively. The mean height was 166 cm ± 6.25 cm, and the body mass index (BMI) was normal (22.27 ± 2.15) according to standard values.

A subgroup of 115 female rowers performed the body composition analysis by bioimpedance. The descriptive analysis of the total sample of female rowers and the subgroup of subjects with body composition analysis is shown in Table 1.

**Table 1:** Descriptive analysis of the total sample of female rowers and the subgroup of subjects with body composition analysis by multifrequency bioimpedance

	Total female rowers sample (n = 208)	Sample with body composition analysis (n = 115)
Age (years)	23.6 ± 6.57	23.7 ± 6.7
Height (cm)	166 ± 6.25	166 ± 6.04
Experience (years)	6.89 ± 4.19	6.72 ± 4.1
Weight (kg)	62.5 ± 7.32	62.5 ± 6.19
BMI (kg/m^2^)	22.7 ± 2.15	22.7 ± 1.92
TBW (L)	–	35.6 ± 3.26
ICW (L)	–	22.2 ± 2.02
ECW (L)	–	13.4 ± 1.26
BFM (Kg)	–	13.8 ± 3.8
FFM (Kg)	–	48.6 ± 4.47
Phase angle-50kHz (º)	–	6.02 ± 0.475

*BMI* body mass index, *TBW* total body water, *ICW* intracellular water, *ECW* extracellular water, *BFM* body fat mass, *FFM* fat free mass

### Measures

Self-administered questionnaires were used to collect information of outcomes in 208 female athletes. Self-reported weight and height were used to calculate BMI.

A subset of 115 female rowers (55% of the total sample) performed the body composition analysis by bioimpedance measurement.

The study was approved by the Ethics Committee of the Ethics Committee of Ramón Llul University (1920005D). All the rowers answered the questionnaire anonymously and accepted informed consent in accordance with the data protection law, Organic Law 3/2018 of 5 December [46], which was attached to the questionnaire.

### Outcomes and procedures

The primary outcomes were the risk of ED symptomatology and the weight-related pressure. Secondary outcome were self-concept, psychological well-being, sociodemographic data, experience, competitive level, and body composition (only in a subgroup of the sample).

#### Questionnaires and competition level

The questionnaire was administered by Google Forms and distributed to all rowing sporting entities and institutions (federations, clubs, and the athletes) of north coast of Spain, where a time limit of 15 days was set to obtain the answers.

The first section of the questionnaire collected the sociodemographic data and performance level of the athletes. Level 1 refers to rowers competing in the highest category named “*Liga Euskotren*” where the best teams from all the north coast can participate. Level 2 refers to the lower leagues such as “*Emakumean Traineru Elkartea”* and “*Liga Gallega de Traineras”* which are allocated in The Basque Country and Galicia, respectively.

Screening for ED symptomatology was assessed by the SCOFF questionnaire [47]. The questionnaire comprises five items and is rated on a dichotomous response scale (yes or no). A score ≥ 2 points was considered as a high risk of ED symptomatology. The Diagnostic and Statistical Manual of Mental Disorders, fourth edition (DSM-IV) criteria for AN and BN also support this conclusion as having satisfactory validity. The questionnaire’s ease of administration stems from its limited number of questions, which, despite their simplicity, effectively probe the major indicators of eating disorders. This is evidenced by Cronbach’s alpha, as each question successfully distinguishes the potentially unhealthy population from the healthy one. The α value for the SCOFF Questionnaire was 0.58, which is acceptable for this purpose [48].

The sport-specific pressures experienced by athletes regarding weight, body shape, size, and appearance was measured using the validated Spanish version of Weight pressures in sport—Females questionnaire (WPS-F) [49, 50]. This has 15 items scores on a six-point Likert scale from 1 (never) to 6 (always). The items in the WPS-F are grouped into two subscales: coach and sport-specific pressures (eight items) and pressures from teammates and due to uniform (seven items) [51]. The Cronbach’s (α) score for the total WPS-F score was 0.864 and the corresponding omega coefficients was 0.873.

The perception of self-concept was measured by the abbreviated version of Physical Self-Concept Questionnaire (CAF-A) [52], which analyses physical self-concept related to physical ability, condition, attractiveness, and strength. The questionnaire’s reliability is demonstrated by a Cronbach’s (α) score of 0.77, as well as subscale reliabilities of strength (0.77), attractiveness (0.83), ability (0.73) and condition (0.66).

Psychological well-being was evaluated by the Spanish version the Ryff scales of Psychological Well-Being [53]. The questionnaire comprises 29 items categorised into self-acceptance, positive relationships, autonomy, environmental mastery, purpose in life, and personal growth. Each subscale’s reliability was Cronbach’s (α) value as follows: self-acceptance (0.83), positive relationships (0.72), autonomy (0.78), mastery of the environment (0.65), purpose in life (0.79), and personal growth (0.66).

#### Body composition

A subgroup of athletes was reported to the laboratory of Deusto University for a single day of testing, which included a body composition assessment. Previously, the completion of questionnaires was checked. Height and weight were initially recorded using a self-calibrating physician’s scale to determine body mass index (BMI). Trained staff used a bioimpedance instrument (InBody 770, Biospace, Korea) to measure body composition was made ≥ 2 h after eating, without exercise, shower or used a sauna before measurements. Athletes were not menstruating and wore shorts while maintaining a standing position with abducted limbs during the measurement conducted in a laboratory with a suitable ambient temperature. They were free of any electronic medical devices. Body composition test was performed holding the hand electrodes with hands such that four fingers covered the bottom electrode while the thumb was positioned on the oval electrode. The heel remained aligned with the rear foot electrode. Participants remained quietly in this position until testing was complete. Body composition variables were body fat mass (BFM), fat free mass (FFM), total body water (TBW), intracellular water (ICW), extracellular water (ECW), and phase angle (PhA).

### Statistical analysis

Descriptive statistics were calculated using the mean as a measure of central tendency and standard deviation as a measure of dispersion. Kolmogorov–Smirnov, Cramer-Von Mises, and Anderson–Darling tests confirmed that the data were normally distributed in all groups.

Several descriptive analyses were set to understand the characteristics of the participants. To study the association between variables, three different χ^2^ Pearson tests were performed (age groups, years of experience groups, and competitive level groups vs. ED symptomatic or non-symptomatic). Cramér’s V was calculated to assess the significance of the association between variables. Cramér’s scale varies from 0 (representing no association between variables) to 1 (perfect association between variables). Pearson’s correlation coefficients were calculated to assess the relationships between variables, using the reference values of < 0.20 very weak, 0.20–0.39 weak, 0.40–0.59 moderate, 0.60–0.79 strong, and > 0.80 very strong. Independent sample Student’s t-test (or its equivalent non-parametric test) was used to detect possible significant differences in descriptive and survey variables between the symptomatic and non-symptomatic ED groups. Cohen’s *d* [54] was performed to study effect size (ES), using the reference values of small (*d* = 0.2), medium (*d* = 0.5) and large (*d* = 0.8). Finally, two multiple regression models were used to predict SCOFF total score and WSP-F total score (dependent variables). All the subscale variables (see outcomes and procedures subsection) were considered as possible predictors for the two regression models. To eliminate possible bias in the interpretation of the regression coefficients, multicollinearity analysis was carried out. Consequently, a matrix of correlations between the independent variables was established. According to previous research [55, 56], a Pearson’s correlation coefficient > 0.8 was related to the likelihood of collinearity. As a result, if there was a strong correlation between two variables (R^2^ > 0.8), one of them was eliminated. The independent variables weight and height were excluded as predictable variables because of their high correlation (R^2^ > 0.8). Following this initial test, the best independent variables for the final regression model were chosen using a stepwise regression method based on Ordinary Least Squares (OLS). The R package olsrr [57] was used to perform these analyses. To identify potential outliers and enhance the regression model’s fit, the dplyr R package was utilized [58]. After outlier detection and stepwise test, each final regression model considered their respective predictable variables. For internal validation, a k-fold cross-validation (10 folds and five repetitions) was performed for each regression model. Internal validation was performed to reduce the possible overfitting of the model [59]. The assumptions of each final regression model were checked (skewness, kurtosis, nonlinear link function, and heteroscedasticity). The root mean square error (RMSE) and R^2^ and R^2^ Adj. were used to evaluate the performance of the model. The model’s error is expressed in outcome units (i.e., points). The significance level for each two-sided statistical analysis was set at p 0.05. R software version 4.3.1 [59] and RStudio version 2023.6.2.561 [60] were used for the statistical analysis.

We recruited 206 subjects without an priori power analysis, thus, we instead conducted three sensitivity analysis in G*Power, which indicated that an effect size of 0.06 for first regression model (Table 4) and 0.07 for the second regression model (Table 5) would be necessary to obtain a power of %80 at an alpha of 0.05 with a regression model based on five (model higher risk of ED symptomatology) and six predictors (model of weight-related pressure). Similarly, the third sensitivity analysis showed the necessity of an effect size of 0.35 to achieving a power of 80% for the Student’s t-test at an alpha of 0.05.

## Results

### Descriptive analysis

The prevalence of high risk of ED symptomatology among female rowers was 42.3% (≥ 2 points in SCOFF) and the mean ± SD value on SCOFF scale was of 1.44 ± 1.28 points. The descriptive analysis of positive responses to the SCOFF items is shown in Supplementary material—Table S1.

The weight-related pressure mean score was 3.65 ± 0.82 points, and 4.01 ± 0.98 and 3.37 ± 0.96 points for the subscales of coach and sport-specific pressures and pressures from teammates and due to uniform, respectively. Total data and questionnaires on self-concept and psychological well-being are shown in Table 2.

**Table 2:** Descriptions of the means of the questionnaire scores

Questionnaire’s responses (n = 208)	Mean ± SD
SCOFF total score	1.44 ± 1.28
WSP-F questionnaire mean of total score	3.65 ± 0.82
WSP coach and sport	4.01 ± 0.98
WSP uniform and teammates	3.37 ± 0.96
CAF total score	25.1 ± 4.47
CAF strength	7.22 ± 1.84
CAF attractiveness	6.18 ± 2.14
CAF ability	4.68 ± 2.14
CAF physical condition	7.00 ± 1.62
Ryff self-acceptance	4.28 ± 0.99
Ryff positive relationships	4.79 ± 0.93
Ryff autonomy	4.02 ± 1.05
Ryff environmental mastery	4.20 ± 0.94
Ryff purpose in life	4.46 ± 1.01
Ryff personal growth	4.98 ± 0.83

*SD* standard deviation, *SCOFF* questionnaire for screening of eating disorders in women, *WSP-F* weight pressures in sport-Females questionnaire, two subscales: coach and sport-specific pressures and Uniform and teammates pressures, *CAF-A* physical self-concept questionnaire abbreviated version; sub variables: ability, condition, attractiveness, and strength. Ryff questionnaire Psychological well-being, with subscale; acceptance, positive relationships, autonomy, environmental mastery, purpose in life, and personal

### Bivariate analysis

Female athletes at high risk of ED symptoms were significantly younger and had higher weight, BMI, levels of weight-related pressure, and poorer self-concept and physical well-being scores (Table 3). There were no statistically significant differences within the most experienced athletes and competition level groups (Supplementary material—Table S2). The analysis of correlations between main and secondary outcomes is shown in the Supplementary material Figs. S1 and S2.

**Table 3:** Differences in athletes with and without eating disorder symptoms

	ED symptomatic (n = 88)	Non-symptomatic (N = 120)	Mean difference	SE of mean difference	*d*
	Mean ± SD	Mean ± SD			
Age (years)	21.76 ± 5.15	24.89 ± 7.17	3.13*	0.9	0.49
Experience (years)	6.26 ± 3.73	7.36 ± 4.45	1.1	0.58	0.26
Height (cm)	165.51 ± 6.43	166.08 ± 6.12	0.56	0.88	0.09
Weight (kg)	63.98 ± 7.91	61.39 ± 6.67	− 2.59*	1.01	− 0.36
BMI (kg/m^2^)	23.34 ± 2.42	22.22 ± 1.78	− 1.11*	0.29	− 0.53
WSP total	3.99 ± 0.76	3.41 ± 0.78	− 0.58*	0.11	− 0.75
WSP Coach and Sport	4.28 ± 0.93	3.82 ± 0.98	− 0.46*	0.13	− 0.47
WSP Uniform and teammates	3.86 ± 0.85	3.02 ± 0.88	− 0.83*	0.12	− 0.96
CAF total	24.2 ± 4.16	25.7 ± 4.59	1.5*	0.62	0.34
CAF strength	7.32 ± 1.87	7.15 ± 1.81	− 0.17	0.26	− 0.9
CAF attractiveness	5.07 ± 1.93	6.99 ± 1.92	1.92*	0.27	1
CAF ability	4.85 ± 2.52	4.56 ± 1.82	− 0.29	0.3	− 0.29
CAF physical condition	6.98 ± 1.60	7.02 ± 1.66	0.04	0.23	0.02
Ryff self-acceptance	3.95 ± 1.02	4.53 ± 0.89	0.58*	0.13	0.61
Ryff positive relationships	4.46 ± 0.88	5.04 ± 0.89	0.58*	0.12	0.65
Ryff autonomy	3.57 ± 3.5	4.36 ± 0.91	0.79*	0.14	0.8
Ryff environmental mastery	3.84 ± 0.95	4.45 ± 0.84	0.61*	0.13	0.69
Ryff purpose in life	4.19 ± 0.98	4.65 ± 0.99	0.43*	0.14	0.47
Ryff personal growth	4.80 ± 0.86	5.12 ± 0.86	0.32*	0.12	0.39

*SD* standard deviation, *SE* standard error, *d* standard deviation, *WSP-F* weight pressures in sport-females questionnaire, *two subscales* coach and sport-specific pressures and Uniform and teammates, *CAF-A* physical self-concept questionnaire abbreviated version, *sub variables* ability, condition, attractiveness and strength; Ryff questionnaire Psychological well-being, with subscale; acceptance, positive relationships, autonomy, environmental mastery, purpose in life, and personal

^*^p value < 0.05

### Regression analysis

After displaying the outlier detection analysis, the final samples for the different regression models were 206 for the total sample and 113 for the body composition subgroup analysis sample.

The multivariable regression model showed that higher physical attractiveness, autonomy, and age were significantly negatively associated with ED symptoms, whereas higher self-perceived strength and pressure from teammates and uniformity were positively associated with ED symptoms (R^2^ Adj. = 0.33; RMSE = 1.04; *p* < 0.05) (Table 4).

**Table 4:** Multiple regression analysis of higher risk of ED symptomatology measured by SCOFF (n = 206)

Predictor	b	b 95% CI [LL, UL]	Beta	Beta 95% CI [LL, UL]	sr2	sr2 95% CI [LL, UL]	t
Intercept	2.50**	[1.49, 3.52]					4.87
CAF attractiveness	− 0.22**	[− 0.30, − 0.14]	− 0.37	[− 0.51, − 0.24]	0.09	[0.03, 0.16]	− 5.36
WSP uniform and teammates	0.03*	[0.01, 0.06]	0.17	[0.04, 0.30]	0.02	[− 0.01, 0.05]	2.55
Ryff autonomy	− 0.04**	[− 0.06, − 0.01]	− 0.18	[− 0.31, − 0.05]	0.02	[− 0.01,0.06]	− 2.72
CAF strength	0.12**	[0.04, 0.21]	0.18	[0.05, 0.30]	0.03	[− 0.01, 0.06]	2.83
Age	− 0.02	[− 0.04, 0.00]	− 0.10	[− 0.23, 0.02]	0.01	[− 0.01, 0.03]	− 1.68

*R*^2^ = 0.35 *R*^*2*^*Adj.* = 0.33** A significant *b*-weight indicates the beta-weight and semi-partial correlation are also significant. *b* represents unstandardized regression weights. *beta* indicates the standardized regression weights. *sr*^*2*^ represents the semi-partial correlation squared. *LL* and *UL* indicate the lower and upper limits of a confidence interval, respectively. * Indicates *p* < 0.05. ** Indicates *p* < 0.01

*WSP* weight pressures in sport-females questionnaire, *two subscales* coach and sport-specific pressures and Uniform and teammates. *CAF-A* physical self-concept questionnaire abbreviated version, *sub variables* ability, condition, attractiveness, and strength, *Ryff* psychological well-being questionnaire, with subscale; acceptance, positive relationships, autonomy, environmental mastery, purpose in life, and personal

The model for the total score of weight-related pressure showed a significant negative association with autonomy and attractiveness, whereas high BMI, athletes’ physical condition, strength, and experience were positively associated with weight-related pressure (R^2^ Adj. = 0.27; RMSE = 12.16; *p* < 0.05) (Table 5). Both models met the assumption criteria of normality and the results of the cross-validation analysis showed no signs of bias.

**Table 5:** Multiple regression analysis of weight-related pressure (n = 206)

Predictor	*b*	*b* 95% CI [LL, UL]	*Beta*	*Beta* 95% CI [LL, UL]	*sr*^2^	*sr*^*2*^ 95% CI [LL, UL]	*t*
Intercept	18.70	[− 2.75, 40.15]					1.72
BMI	1.91**	[1.02, 2.81]	0.28	[0.15, 0.42]	0.06	[0.01, 0.12]	4.23
Ryff autonomy	− 0.47**	[− 0.78, − 0.17]	− 0.21	[− 0.34, − 0.07]	0.03	[− 0.01, 0.08]	− 3.07
CAF condition	1.93**	[0.66, 3.21]	0.22	[0.07, 0.36]	0.03	[− 0.01, 0.07]	2.99
CAF attractiveness	− 1.58**	[− 2.56, − 0.61]	− 0.23	[− 0.38, − 0.09]	0.04	[− 0.01, 0.08]	− 3.20
Experience	0.53*	[0.11, 0.95]	0.15	[0.03, 0.28]	0.02	[− 0.01, 0.06]	2.47
CAF strength	1.06	[− 0.11, 2.24]	0.14	[− 0.01, 0.29]	0.01	[− 0.01, 0.04]	1.79

*R*^*2*^ = 0.29** R^*2*^* Adj.* = 0.27. A significant *b*-weight indicates the beta-weight and semi-partial correlation are also significant. *b* represents unstandardized regression weights. *beta* indicates the standardized regression weights. *sr*^*2*^ represents the semi-partial correlation squared. *LL* and *UL* indicate the lower and upper limits of a confidence interval, respectively. *Indicates *p* < 0.05. ** Indicates *p* < 0.01

*BMI* body mass index, *Ryff* psychological well-being questionnaire, *CAF* physical self-concept questionnaire abbreviated version, *BMI* body mass index

### Regression analysis for the subgroup of body composition

In the multivariable models performed on the subset of 113 athletes who underwent bioimpedance analysis, no associations were found between body composition variables and ED symptoms. In the weight-related pressure model, a higher level of ECW was associated with weight pressure, in addition to self-concept and physical well-being outcomes (Supplementary material S3A, B).

## Discussion

This study found, for the first time, that fixed-bench female rowers were at high risk of ED symptoms and perceived weight-related pressure. In addition, athletes’ self-concept of strength and pressure from teammates and the uniform were associated with higher ED symptoms, whereas better psychological well-being in terms of autonomy, self-concept of attractiveness, and age were protective factors for ED symptoms. For weight-related pressure higher BMI, athletes’ physical condition, strength, and experience were associated with more pressure, whereas better self-concept of attractiveness and physical well-being of autonomy were significantly associated with less pressure. Body composition data measured by bioimpedance showed no association with ED symptoms. However, higher ECW, self-acceptance, and physical condition were associated with more weight-related pressure in female rowers, being attractiveness and the environmental mastery protective elements.

### Prevalence of ED symptomatology and regression analysis

Our findings showed that 42.3% of female fixed-bench rowers were at risk for ED symptoms according to the SCOFF scale. These findings are consistent with previous research in other rowing modalities. Amon Olympic women rowers, high levels of risky weight regulation behaviours and ED attitudes have been consistently reported [31], particularly among lightweight rowers [14, 42]. However, the mean of SCOFF data of obtained in fixed-bench rowing is higher than other rowing modalities [31]. Likewise, the prevalence of ED symptoms is higher than showed in women lightweight rowers [42] and the rates reported in the largest multi-sports study to date carried out in Spain [51].

Indeed, female athletes with high risk of ED symptoms showed higher weight-related pressure from teammates and due to uniform. In general, it is known that pressure to achieve or keep a low body weight and/or a lean body shape is considered an important risk factor for ED symptoms [18, 61, 62], especially in aesthetic sports such as rowing, due to the athletes’ perception that weight or body shape significantly affects performance [18, 28]. Our finding is consistent with other studies that have found that female athletes with ED symptoms score higher on the weight pressure scale than those without ED symptoms [28, 63–65]. However, the focus of the analysis should be on the type of pressure. Teammates and due to the uniform pressure showed an association with ED symptoms in female fixed-bench rowers, whereas coach and sport-specific pressure did not. In the fixed bench rowing, the teams are made up of 14 athletes and with the substitutes they form crews of about 22 athletes. We hypothesize that this number of athletes is noteworthy as there may be more competition and pressure between athletes. In addition, as an aerobic sport, they spend a great deal of time in each other’s company during daily training sessions. In this regard, previous studies have reported that the interaction with teammates can lead to unhealthy attitudes due to negative comments from teammates about weight or size that can trigger body image concerns or restrictive eating behaviours, especially in female athletes [23, 67, 68]. A recent systematic review found that teammates can negatively influence athletes’ eating attitudes through four mechanisms described as conflicting friendships with teammates, critical comments and conversations about appearance, maladaptive team norms, and competitive comparisons with teammates [23]. Moreover, it is known that athletes may also experience pressure when information regarding body weight is made publicly available via team weigh-ins [21] or when their body is presented in a uniform in addition to their performance [68]. In this regard, various studies in cheerleaders, swimmers and dancers have found that the uniform may be a factor related to weight pressure if it has a shape that may reveal bodily flaws [49, 66, 69, 70]. Although rowing is not an aesthetic sport, athletes wear revealing uniforms in training as well as in competition, and are viewed by spectators, which may increase unhealthy body perceptions and accentuate aesthetic imperfections and subjective judgements, a combination that may serve to increase the risk of developing DE behaviours.

In turn, athletes’ high self-concept of strength (perceived as feeling strong, able to lift weight, and predisposition toward exercise) was associated positively with ED symptoms. This is consistent with previous research on the immersion of elite athletes in a discipline within a highly competitive, physically demanding, weight-conscious culture. This may encourage and emphasise thinness and leanness to maximise performance or meet aesthetic ideals [71], through physical overtraining in terms of time, intensity, or load, commonly attributed to endurance sports [21, 23].

On the other hand, athletes with better autonomy and self-concept of physical attractiveness were less susceptible of ED symptoms. These results support the etiological model to explain the development of DE among athletes of Petrie and Greenleaf which includes self-concept and well-being factors as mediators [14], which are protective in the case of female fixed-bench athletes. Moreover, it is known that positive body image appreciation, self-esteem, self-compassion, and psychological well-being are protective factors for DE in general female population [11, 13] as well as in athletes [12]. In turn, older age was associated with reduced ED symptoms. This is consistent with recent research that has been shown that adolescent competitive female athletes reported more frequent self-induced vomiting, laxative misuse, and excessive exercise, as well as poorer healthy eating habits, compared with adult female athletes [12]. In fact, adult female athletes have significantly more positive body image perceptions than adolescents, which may be related to better self-concept and physical well-being, as mentioned above [13]. We hypothesize that this novel finding may be related to the rowers’ emotional regulation ability strategies such as reappraisal, body image perception, or active problem-solving strategies based on awareness and acceptance of emotions, attitudes that are more appropriate to an adult population [72]. However, as the authors acknowledge, there is a need to test the impact of these factors and the negative or positive trend, mediation, and associations between them.

Overall, the psychological well-being and self-concept may play an important role in protecting athletes against the development of ED symptoms, and subsequent disturbed attitudes and behaviours. Although there has been very limited longitudinal exploration on this topic, this finding highlights an important target for future intervention programmes aimed at preventing unhealthy behaviours in female fixed-bench athletes. In light of this, more research is needed on the key protective/facilitating factors against the development of DE, to facilitate the identification of high-risk groups, to tailor prevention and intervention programmes for athletes, coaches, sports professionals, physical trainers and parents, and to ensure that sports policies include health professionals (psychologists, nutritionists or physiotherapists) who are aware of ED among technical staff.

### Weight-related pressure and regression analysis

The perceived weight-related pressure data in fixed-bench female rowers with ED symptomatology is substantially higher comparing with other sports such as female adolescent gymnasts or the large Spanish cohort of elite athletes from 33 sports with or without ED symptoms [3, 66]. We hypothesise that the general goal of reducing the weight of the boat and properly distributing the crew to achieve greater team performance in terms of boat speed [35] promotes collective concern and an increase in weight-related pressure, resulting in a higher risk of ED symptoms [2].

In addition, our findings support that athletes’ high experience, self-concept of physical condition, strength, and BMI were associated with more weight-related pressure, which is consistent with previous studies showing a higher weight-related pressure in higher-level or competitive athletes [65, 66]. On the contrary, athletes with better autonomy and attractiveness were significantly protected against weight-related pressure. This is in line with previous research that has shown that greater body esteem means that female athletes express respect and love for their bodies and are more resilient to socio-cultural pressures against stereotypical body ideals [12, 13, 73].

In body composition analysis measured by bioimpedance ECW showed an association with perceived weight-related pressure together with self-acceptance and physical condition dimensions. Few studies have focused on body composition and showed that ED symptoms were more likely in female athletes with a higher BMI in different sports [66, 74]. However, in this and other rowing studies, BMI was not associated as a risk factor for ED symptoms [31], but it did show an association with weight pressure. For our knowledge, this is the first study that showed an association between high values of ECW and weight-related pressure. This may be related to water retention associated with the electrolyte balance, hormone sensitivity and first days of menstrual cycle in female athletes which may increase the concerns on body image perception, but further research is warranted [75].

The current study has some limitations. The cross-sectional design did not allow for cause and effect to be determined, meaning that longitudinal and experimental studies are required to determine temporal relationships during and after competitive season. The SCOFF scale should be complemented by other ED questionnaires to obtain more specific information regarding to ED psychopathology. It would be interesting to include male athletes in future studies and to analyse the influence of the coach. Likewise, no parameter was included to determine the degree of training or energy intake and the relatively small size of the sample also may be a limitation. The main strength of this study are the inclusion of self-concept and psychological well-being questionnaires and the measurement of bioimpedance. In addition, the sample is made up of 45% of the total number of athletes practising this sport in the Cantabrian Sea in an official and federated way.

## Conclusion

Female fixed-bench rowers were at high risk of ED symptoms and perceived weight-related pressure. Teammates and the uniform pressure and self-concept of strength were associated with higher ED symptoms in female rowers, whereas better self-concept, psychological well-being, and more age showed less susceptibility of ED symptoms. Higher BMI, experience, self-concept of physical condition, and strength were associated with more weight-related pressure, whereas better self-concept of attractiveness and physical well-being of autonomy were significantly associated with less pressure. From body composition analysis only ECW was associated with more weight-related pressure in female rowers. The psychological factors of self-concept, well-being and body composition analysis should be considered to identify the risk of ED symptoms and weight pressure in female fixed-bench rowers, which may help to design more targeted prevention strategies for this vulnerable athlete population. More research is needed on the key protective/facilitating factors against the development of DE in order to tailor prevention and intervention programmes for athletes, coaches, parents and technical staff, and to ensure that sports policies include health professionals (psychologists, nutritionists or physiotherapists) who are aware of ED.

### Supplementary Information

Supplementary Material.

## Data Availability

The datasets generated and/or analysed during the current study are not publicly available due the main reason for certain data not being public is the presence of sensitive information that, if disclosed, could compromise individuals' privacy and security but are available from the corresponding author on reasonable request.

## Abbreviations

AN: Anorexia nervosa
BFM: Body fat mass
BMI: Body mass index
BN: Bulimia nervosa
CAF-A: Physical self-concept questionnaire
d: Standard deviation
ECW: Extracellular water
ED: Eating disorder
ES: Effect size
FFM: Fat free mass
ICW: Intracellular water
OLS: Ordinary least squares
PhA: Phase angle
RMSE: Root mean square error
SCOFF: Questionnaire for screening of eating disorders in women
SD: Standard deviation
SE: Standard error
TBW: Total body water
WPS-F: Weight pressures in sport-females questionnaire
