# Supplementary material for: Eating disorder symptoms and weight pressure in female rowers: associations between self-concept, psychological well-being and body composition
Source: J Eat Disord. 2024 Jun 14;12:81. doi: 10.1186/s40337-024-01033-9 (PMC11177466; doi:10.1186/s40337-024-01033-9)
Supplement: Supplementary file 1 — Supplementary Material. [file 40337_2024_1033_MOESM1_ESM.docx]

**Supplementary material**

*Table S1.* *Descriptive analysis of positive responses to the SCOFF items.*

| SCOFF Items | n | % |
| --- | --- | --- |
| 1.Vomiting, yes (%) | 11 | 5.29 |
| 2. Worry about losing control, yes (%) | 117 | 56.25 |
| 3. Lost more than 6 kg weight, yes (%) | 33 | 15.87 |
| 4. Belive yourself to be fat, yes (%) | 61 | 29.33 |
| 5. Food dominates life, yes (%) | 76 | 36.54 |

*Note*: SCOFF; questionnaire for screening of eating disorders in women.

*Table S2.* *Differences in age, experience, and performance level between athletes with and without eating disorder symptoms.*

| n=208 | ED symptomatic | | Non-symptomatic | | X² | p-value | Cramér’s V |
| --- | --- | --- | --- | --- | --- | --- | --- |
| Age (years) | n | (%) | n | (%) |  |  |  |
| <18 (n= 19) | 12 | 63.2 | 7 | 36.8 | 13.4 | 0.01 | 0.25 |
| 18-25 (n=118) | 57 | 48.3 | 61 | 51.7 |  |  |  |
| 26-30 (n=44) | 14 | 31.8 | 30 | 68.2 |  |  |  |
| 31-35 (n=12) | 3 | 20.0 | 12 | 80.0 |  |  |  |
| >35 (n=10) | 2 | 16.7 | 10 | 83.3 |  |  |  |
| Years of experience (years) | | | | | | |  |
| 1-5 (n=88) | 45 | 51,1 | 43 | 48.9 | 6.08 | 0.11 | 0.17 |
| 5-10 (n=78) | 29 | 37.2 | 49 | 62.8 |  |  |  |
| 10-15 (n=28) | 11 | 39.3 | 17 | 60.7 |  |  |  |
| >15 (n=14) | 3 | 78.6 | 14 | 21,4 |  |  |  |
| Competitive level | | | | | | | |
| Level 1 (n=45) | 22 | 48.9 | 23 | 51.1 | 1.02 | 0.31 | 0.07 |
| Level 2 (n=163) | 66 | 40.5 | 97 | 59.5 |  |  |  |
| Total | | | | | | | |
|  | 88 | 42.3 | 120 | 57.7 |  |  |  |

*Note:* ED; psychopathology or behaviour to a clinical diagnosis of an eating disorder.

| Predictor | *b* | *b*  95% CI  [LL, UL] | *beta* | *beta*  95% CI  [LL, UL] | *sr^2^* | *sr^2^*  95% CI  [LL, UL] | *r* |
| --- | --- | --- | --- | --- | --- | --- | --- |
| Intercept | 61.21** | [34.81, 87.61] |  |  |  |  |  |
| Ryff autonomy | -0.75** | [-1.20, -0.31] | -0.32 | [-0.51, -0.13] | 0.07 | [-0.01, 0.15] | -0.39** |
| ECW | 2.13* | [0.35, 3.90] | 0.19 | [0.03, 0.35] | 0.03 | [-0.02, 0.09] | 0.14 |
| CAF physical condition | 1.97** | [0.49, 3.45] | 0.22 | [0.05, 0.39] | 0.04 | [-0.02, 0.10] | 0.04 |
| CAF attractiveness | -3.02** | [-4.69, -1.34] | -0.42 | [-0.65, -0.19] | 0.08 | [-0.00, 0.16] | -0.40** |
| Ryff environmental mastery | -0.92* | [-1.71, -0.13] | -0.25 | [-0.46, -0.04] | 0.03 | [-0.02, 0.09] | -0.37** |
| Ryff self-acceptance | 0.95 | [-0.06, 1.96] | 0.26 | [-0.02, 0.53] | 0.02 | [-0.02, 0.06] | -0.32** |
| *Note.* *R^2^*  =0 .34**  *R^2^* Adj. = 0.30**; RMSE = 11.42. A significant *b*-weight indicates the beta-weight and semi-partial correlation are also significant. *b* represents unstandardized regression weights. *beta* indicates the standardized regression weights. *sr^2^* represents the semi-partial correlation squared. *r* represents the zero-order correlation. *LL* and *UL* indicate the lower and upper limits of a confidence interval, respectively. * indicates *p*<0.05. ** indicates *p* < 0.01. *ECW:* Extracellular water; WSP-F: Weight pressures in sport - Females questionnaire; Ryff: Psychological well-being questionnaire. CAF: Physical Self-Concept Questionnaire abbreviated version. | | | | | | | |

| Predictor | *b* | *b*  95% CI  [LL, UL] | *beta* | *beta*  95% CI  [LL, UL] | *sr^2^* | *sr^2^*  95% CI  [LL, UL] |
| --- | --- | --- | --- | --- | --- | --- |
| Intercept | 3.13** | [1.56, 4.70] |  |  |  |  |
| Ryff environmental mastery | -0.11** | [-0.16, -0.05] | -0.32 | [-0.50, -0.14] | 0.07 | [-.00, 0.14] |
| CAF attractiveness | -0.24** | [-0.35, -0.13] | -0.39 | [-0.57, -0.21] | 0.10 | [0.01, 0.19] |
| CAF strength | 0.16** | [0.05, 0.28] | 0.22 | [0.06, 0.37] | 0.04 | [-0.01, 0.10] |
| CAF ability | -0.14** | [-0.23, -0.05] | -0.25 | [-0.40, -0.09] | 0.05 | [-0.01, 0.12] |
| WSP uniform and teammates | 0.03* | [0.00, 0.05] | 0.17 | [0.02, 0.32] | 0.03 | [-0.02, 0.07] |
| *Note.* *R^2^* = 0.42** *R^2^* Adj. = 0.39**A significant *b*-weight indicates the beta-weight and semi-partial correlation are also significant. *b* represents unstandardized regression weights. *beta* indicates the standardized regression weights. *sr^2^* represents the semi-partial correlation squared. *r* represents the zero-order correlation. *LL* and *UL* indicate the lower and upper limits of a confidence interval, respectively. * indicates *p*<0.05. ** indicates *p*<0.01. WSP-F: Weight pressures in sport - Females questionnaire. Ryff: Psychological well-being questionnaire. CAF: Physical Self-Concept Questionnaire abbreviated version. | | | | | | |

*Table S3-A. Multiple regression analysis of higher risk of ED symptomatology measured by SCOFF in the sample with body composition analysis measured by bioimpedance (n=113).*

*Table S3-B. Multiple regression analysis of weight-related pressure in the sample with body composition analysis measured by bioimpedance (n=113)*

Figure S1:

*Pearson Correlation Plot of primary and secondary outcomes in all sample (n=208).*


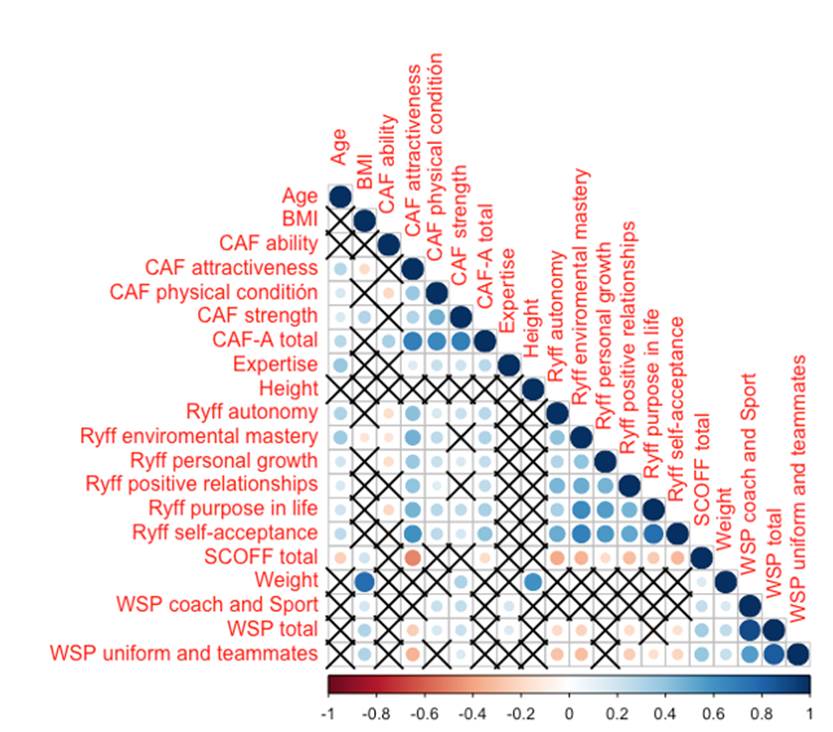


*Note:* X = *p* > 0.05.

BMI: Body mass index. WSP; Weight pressures in sport - Females questionnaire; Ryff: Psychological well-being questionnaire. CAF: Physical Self-Concept Questionnaire abbreviated version.

 Figure S2:

*Pearson Correlation Plot of primary and secondary outcomes in subset sample with body composition analysis (n=115).*


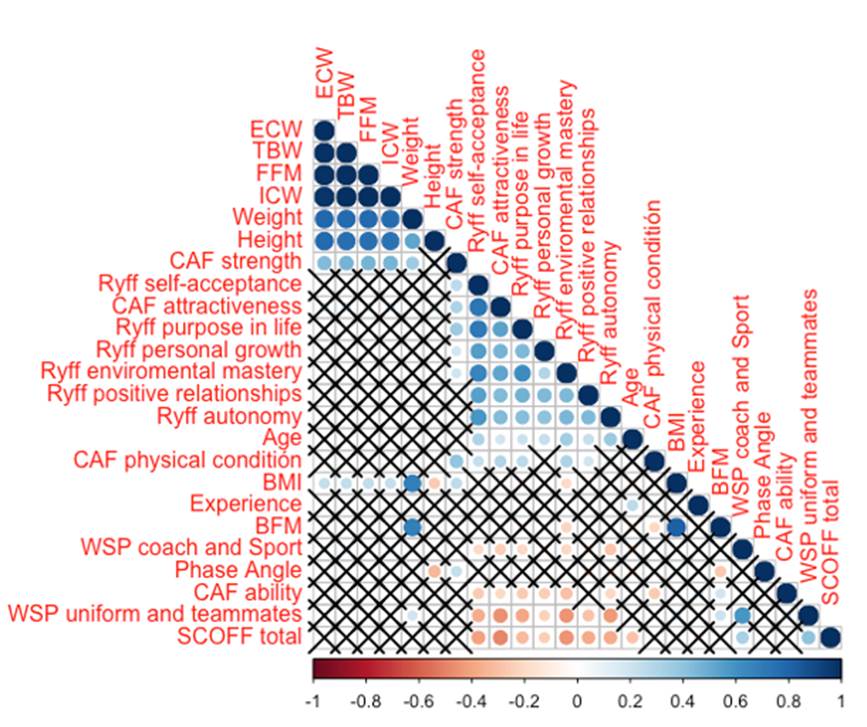


*Note:*  X = *p* > 0.05.

ECW: Extracellular Water; TBW: Total Body Water; Body Fat Mass; FFM: ICW: Intracellular Water; BMI: Body mass index; TBW: Total Body Water; ICW: Intracellular Water; ECW: Extracellular Water; BFM: Body Fat Mass; FFM: Fat Free Mass; WSP: Weight pressures in sport - Females questionnaire; Ryff: Psychological well-being questionnaire. CAF: Physical Self-Concept Questionnaire abbreviated version. SCOFF: questionnaire for screening of eating disorders in university women.
